# Automated *In-Situ* Laser Scanner for Monitoring Forest Leaf Area Index

**DOI:** 10.3390/s140814994

**Published:** 2014-08-14

**Authors:** Darius S. Culvenor, Glenn J. Newnham, Andrew Mellor, Neil C. Sims, Andrew Haywood

**Affiliations:** 1 CSIRO Land & Water, Private Bag 10, Clayton South, VIC 3169, Australia; E-Mail: neil.sims@csiro.au; 2 Environmental Sensing Systems, 16 Mawby Road, Bentleigh East, VIC 3165, Australia; E-Mail: darius.culvenor@sensingsystems.com.au; 3 Victorian Department of Environment and Primary Industries, 8 Nicholson Street, East Melbourne, VIC 3002, Australia; E-Mails: andrew.mellor@depi.vic.gov.au (A.M.); andrew.haywood@depi.vic.gov.au (A.H.)

**Keywords:** Lidar, rangefinding, forest, LAI, canopy, VEGNET

## Abstract

An automated laser rangefinding instrument was developed to characterize overstorey and understorey vegetation dynamics over time. Design criteria were based on information needs within the statewide forest monitoring program in Victoria, Australia. The ground-based monitoring instrument captures the key vegetation structural information needed to overcome ambiguity in the estimation of forest Leaf Area Index (LAI) from satellite sensors. The scanning lidar instrument was developed primarily from low cost, commercially accessible components. While the 635 nm wavelength lidar is not ideally suited to vegetation studies, there was an acceptable trade-off between cost and performance. Tests demonstrated reliable range estimates to live foliage up to a distance of 60 m during night-time operation. Given the instrument's scan angle of 57.5 degrees zenith, the instrument is an effective tool for monitoring LAI in forest canopies up to a height of 30 m. An 18 month field trial of three co-located instruments showed consistent seasonal trends and mean LAI of between 1.32 to 1.56 and a temporal LAI variation of 8 to 17% relative to the mean.

## 1. Introduction

Leaf Area Index (LAI) is one of the most informative attributes used by the ecological community for monitoring forest function. Defined as half the area of green leaves per unit ground area [1], LAI influences the interception of solar radiation and characterizes the interface between the canopy and the atmosphere [2]. It is strongly related to plant productivity [3] and is the primary variable used in many canopy process models [4,5]. LAI was recommended in the 1998 IPCC report on regional vulnerabilities to climate change [6] as a promising indicator of climate change impacts on vegetation.

Recognizing the importance of LAI as part of the global carbon cycle, a number of spatial LAI estimates are available as standard earth-observation products. For example, the MODIS sensor on the Terra EOS satellite observes the Earth at a spatial resolution from 250 m to 1 km and a temporal resolution of 1–16 days. Daily MODIS observations are used to produce a composited 8-day global LAI product at a spatial resolution of 1 km. However, the accuracy and consistency of these products are difficult to validate with existing methods.

The downward-looking orientation of earth-observation sensors results in an influence of both overstorey and understorey vegetation on estimates of LAI. It is known that spectral influences from soil background and understorey vegetation can introduce uncertainty in satellite-based estimates of overstorey LAI, particularly in forests with low canopy cover, e.g., [7–9]. Variations in overstorey and understorey vegetation over time further confound current validation techniques. Understanding and addressing variability from all of these sources can only be achieved through quantitative, *in-situ* measurement of these strata at a temporal scale compatible with earth-observation satellite data. One way to achieve this is through a network of ground-based sensors designed to directly measure, through active rangefinding, the vertical distribution of plant material. It is in this context that a new type of ground calibration instrument has been developed.

This paper describes a low cost *in-situ* scanning lidar instrument for monitoring vertical vegetation structure on a daily timescale. In contrast to most terrestrial laser scanners, an *In-situ* Monitoring Lidar (IML) is designed for automated (unattended) operation and semi-permanent installation at a site. This requires consideration of reliability, weather-proofing, power consumption and data volume. The design criteria and decisions for a new IML instrument are explained, and results from an 18-month field trial are presented.

## 2. Instrument Description

### 2.1. Design Criteria

Laser rangefinding is an effective means of characterizing the vertical structure of vegetation, including ground-based instruments e.g., [10–12] and airborne laser scanners e.g., [13,14]. While a possible solution to decoupling overstorey and understorey dynamics is to deploy multiple passive sensors at different heights in the canopy, this presents practical problems in forests with very tall understorey, and requires an *a priori* decision about the height of important structural transitions within the canopy. Given that vegetation can be structurally diverse, a monitoring instrument needs to be capable of recording distance to targets from as close as 0.5 m to upper canopy surfaces at distances of 70–100 m.

Additionally, to allow cost effective networks of instruments to be installed over large areas it was considered important that the IML be constructed mostly from low cost, commercially accessible components. A maximum cost of USD $5000 was used to guide the design and selection of hardware.

The intended application of the IML as a long-term monitoring device requires a robust design to withstand harsh environmental conditions (rain, wind, humidity and extremes of temperature). Simple construction with few moving parts were additional objectives to keep power consumption, failure rate and data volume to a minimum.

**Figure 1.**
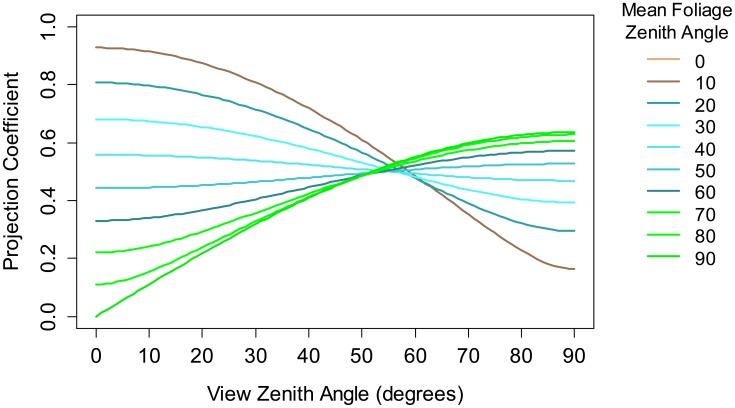
The projected coefficient for leaf area estimation according to the Campbell model [16] for mean leaf surface normal zenith angles ranging from zero degrees (brown) to ninety degrees (green).

The IML instrument was designed primarily as a monitoring tool with wide spatial coverage to reduce LAI estimation error attributable to local spatial variability. This requirement introduced the need for scanning functionality so that the laser could be directed to sample different parts of the canopy from a fixed location. A means of achieving this “plot scale” sampling was to implement variable azimuth and fixed zenith angle (almucantar) scanning. A single data acquisition sequence therefore requires only one full-azimuth sweep of the laser. The zenith angle of the laser was chosen as 57.5°, otherwise known as the “hinge” angle [10]. LAI estimation from angular sampling is dependent on an assumption of randomly distributed leaves in the horizontal plane and knowledge of the leaf angle distribution (LAD). However, the sensitivity of LAI estimation to the leaf angle distribution is low at a view zenith angle of 57.5° [15] as illustrated in Figure 1.

**Figure 2.**
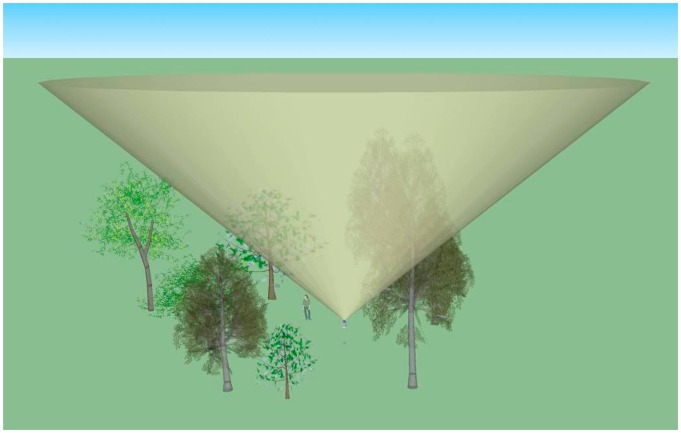
Model of *In-situ* Monitoring Lidar (IML) scan geometry depicting 0–360° azimuth scan at a constant zenith angle of 57.5°.

Figure 2 shows the scan geometry of the IML, represented by a full azimuth scan at a zenith angle of 57.5°. The canopy volume sampled by the laser is defined by laser beam diameter, beam divergence and range. The scan zenith angle also defines the effective scan radius. In a forest with uniform canopy height the IML laser will exit the canopy at a horizontal distance from the instrument being twice the canopy height. The IML instrument would therefore sample an area of 1 hectare if established in a forest with a canopy height of 28 m.

### 2.2. Instrument Description

Few commercially available rangefinders are capable of measuring across the wide dynamic range required for *in-situ* vegetation monitoring (0.5 m–100 m). After comparing manufacturer specifications for various rangefinders, a Bosch GLM150 was selected as a good compromise between cost and performance. Bosch specifies the GLM150 as having a ranging capability of (5 cm–150 m) with an accuracy of ±1 mm. Once triggered, a range operation takes between 0.5 and 4 s depending on the strength of the return signal. Designed primarily for the building construction and home renovator markets, the GLM150 is a single-return, phase-based ranging instrument intended for manual handheld operation. While this instrument is relatively inexpensive, it presents two main compromises: first, the GLM150 does not have a computer interface, and second, the laser wavelength of 635 nm is not ideal for vegetation monitoring due to strong absorption of red light by chlorophyll. Tests performed on low cost red wavelength rangefinders (including the GML150) determined that night-time operation was required to detect a sufficiently strong signal from live foliage for valid ranging at operationally useful distances (>30 m).

An Atmel XMega128A1 (XMEGA) microcontroller is used to control all operations of the IML sensor head. An interface between the XMEGA and the GLM150 was developed to enable automated triggering of the rangefinder and recording of range measurements. While all range data are available at millimeter resolution, far field measurements are not required at such high precision for forest monitoring. Consequently, range data are stored on a microSD memory card at millimeter precision for distances <10 m and centimeter precision for distances ≥10 m. Gaps in the canopy or erroneous range measurements (e.g., due to low signal or target movement) are reported as a range of 0.000 m. Results of all range measurements are recorded irrespective of whether the value represents a valid target or a gap in the canopy.

**Figure 3.**
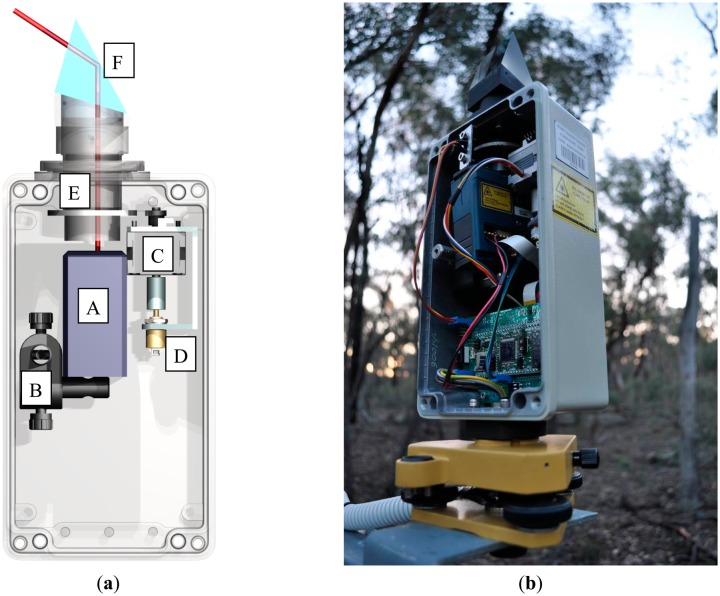
(**a**) Model of *In-situ* Monitoring Lidar (IML). Components are identified as: GLM150 rangefinder (A), laser mount and alignment (B), stepper motor (C), rotary encoder (D), rotary shaft (E), scan head (F) and laser optical path (red line). (**b**) IML sensor head.

Scanning action of the IML is provided by a stepper motor that drives a hollow rotary shaft. Mounted on top of the shaft is an inclined 45° prism with anti-reflective coating, referred to hereafter as the “scan head”. The GLM150 laser beam is directed vertically through the rotary shaft and subsequently reflected by the scan head at a zenith angle of 57.5°. The optical path of the VEGNET IML is illustrated in Figure 3a. The scan head is stationary during range measurements. Return energy is reflected back through the rotary shaft to the receive aperture of the GLM150. A magnetic absolute rotary encoder (US Digital model MA3) records the azimuth angle of the scan head to a precision of 0.1° and accuracy <0.5°. A slotted optical switch and anti-backlash gears between the stepper motor and rotary shaft ensure directional consistency between scans. Laser optical alignment is achieved by mounting the GLM150 to its enclosure using two optical posts and a right-angle clamp.

Figure 3b shows the IML sensor head. All sensing components except for the scan head are housed within an aluminum enclosure with an IP67 ingress protection rating (protection against dust and heavy rain). The rotary shaft protrudes from the enclosure through a sealed stainless steel and ceramic bearing. The enclosure is attached to a Zeiss style survey tribrach for precise three-axis levelling. A power cable passes through a gland seal at the base of the enclosure. Power is supplied by four 6 V 12 Ah sealed lead-acid (SLA) batteries and a 15 W solar panel. The batteries are housed in an enclosure separate from the sensor head. Longevity of the batteries and the effectiveness of solar recharging are dependent on canopy density, topography and latitude.

The battery enclosure includes an Atmel ATMEGA 1281 microcontroller module with a real time clock (RTC) and solid state memory, a GPS for periodic time synchronization, a relay switch for applying power to the sensor head, and a 5 A solar charge regulator for charging the SLA batteries. The battery and sensor head enclosures are connected by a four-core cable supplying power and serial communications.

The IML can be programmed to acquire up to 7360 range measurements for a full azimuth scan (an average of 20.6 measurements per azimuth degree). A typical scan configuration of 920 measurements (2.6 per degree) takes approximately 40 min to complete. Scan time is longer in sparse canopies because the GLM150 will sample canopy gaps for up to four seconds before it abandons the measurement. Optimum scan density will depend on forest structure and power availability. The IML draws approximately 4 Watts of power during operation, mostly by the stepper motor. The duty cycle of the GLM150 is also a consideration when defining scan density. The duty cycle is not reported by Bosch but it is reasonable to assume that the GLM150 is not designed for continuous automated operation. It is therefore prudent to minimize scan density where possible.

A data file from the IML at a scan density of 920 “shots” per scan is 28 KB uncompressed. This enables data files from long term monitoring to be easily accessed via wireless sensor networks or cellular phone link. While the IML instruments can be operated as independent monitoring tools, each instrument has a wireless transceiver so that they can collectively form a vegetation monitoring network referred to as “VEGNET”. The VEGNET concept aims to increase the objectivity and temporal frequency of *in-situ* forest structural monitoring for various applications, but particularly for the calibration and improved interpretation of earth-observation data.

### 2.3. Data Analysis

Processing of IML data first requires calculating the height *h* above ground for all measured objects. Assuming flat ground, height *h* is calculated as the cosine of the laser zenith angle (57.5°) and the laser distance measurement *d*:

(1) h=cos(57.5°)d

Vegetation vertical structure is subsequently described in terms of canopy gap probability (Pgap). For the IML, Pgap at height *h* in the canopy is a simple ratio of the number of valid returns below *z* (#*z_i_* < *h*) to the total number of laser shots (*N*) such that:

(2) $$\text{Pgap}(z)=\left[\#z_{i}<h\right]/N$$

The conversion of Pgap(*z*) to a cumulative LAI up to height *h* uses an extension of the Monsi and Seaki equation [17] often applied in the analysis of hemispherical photography. In the case of IML, LAI is estimated as a cumulative profile from instrument height (*z* = 0) to a height *z* = *h* as specified by [10] for the hinge angle using the equation:

(3) LAI(z)=−1.1×ln(Pgap(z))

The density of vegetation components at any level *z*, sometimes referred to as the plant area volume density (PAVD), is then computed as the derivative of LAI with respect to height as follows:

(4) PAVD(z)=δLAI(z)/δz

Estimation of LAI from Pgap does not differentiate stems, branches and foliage [18], hence LAI in this instance is more correctly termed the Plant Area Index (PAI), e.g., [19].

## 3. Field Trial

### 3.1. Study Area

**Figure 4.**
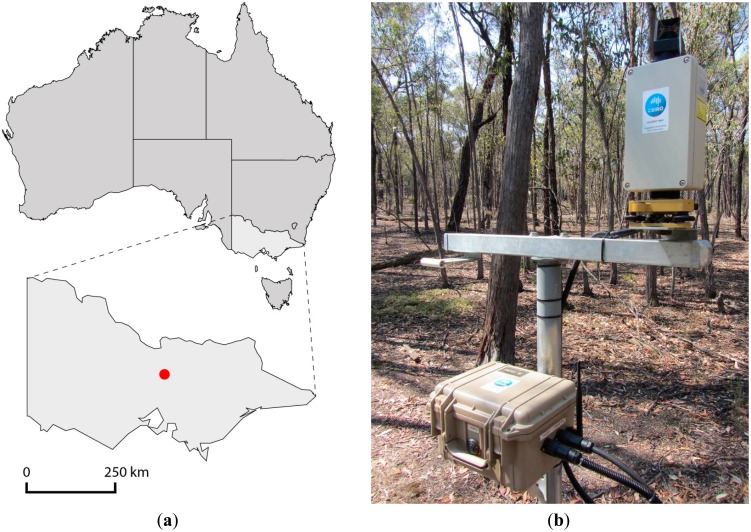
(**a**) Field site location (red circle) in the State of Victoria, Australia. (**b**) Picture of IML instrument number VN5 at the field site. The cross-bar mounting structure enables future co-location of both upward- and downward scanning IML instruments.

A field trial was established in a mixed-species dry sclerophyll eucalypt forest in Central Victoria, Australia (37.7° S, 144.9° E), Figure 4a. The forest type is formally classified as Eucalypt Open Forest, typified by an open canopy and a height of 10–30 m [20].

Three VEGNET IML instruments (labelled VN5, VN6 and VN7) were deployed at the vertices of an equilateral triangle with a separation distance of 50 m. The instruments were mounted on secure steel poles 1.5 m above the ground as shown in Figure 4b.

The study area has a southerly aspect with a gentle slope of approximately 5%. Instrument number VN5 was located in a mid-slope position, VN6 at an upper slope position and VN7 in a lower slope position. A weather station was located near the center of all three IML instruments. The Davis Vantage Vue^®^ weather station recorded rainfall (0.2 mm increments), temperature, relative humidity, wind speed, wind direction and barometric pressure at 2 min intervals.

The field site was visited at least monthly to ensure instruments were well maintained. Key maintenance tasks were to clean the exposed optical surface of the scan heads, calibrate the sensor real time clocks and clean solar panels. The weather station was also checked to ensure the rain gauge was free of debris and anemometer was rotating freely.

### 3.2. Data Filtering

The microcontroller in the battery enclosure initiates a VEGNET IML scan daily at 10 PM local time. Some of the scans will inevitably be acquired under adverse weather conditions (rain and/or high wind). The external surface of the scan head prism is deliberately exposed to rainfall. This keeps the prism surface relatively clean from dust, but most importantly, minimizes insect and spider habitation of the prism face. Droplets of rain on the scan prism scatter and reflect laser energy resulting in near-field range measurements of approximately 270 mm, which is the distance from the rangefinder to the scan prism outer surface. Consequently, rain-affected scans are easily identifiable in the time-series data as having consistently low range measurements. To conserve power, scans are automatically terminated following 50 consecutive range measurements less than 300 mm.

Wind-affected scans are less easily identifiable during post-processing. The availability of local weather records assists with the selection of good quality scans. Thresholds for weather-based data filtering may be defined effectively for different study areas through empirical comparison of cumulative PAI and PAVD profiles under different weather conditions. In cases where some scan data are deemed weather-affected, the whole scan must be excluded to avoid introducing directional bias in the forest structural estimates. Inclusion of partial scans would otherwise increase the weight of included azimuth ranges in the overall PAI and PAVD results.

**Figure 5.**
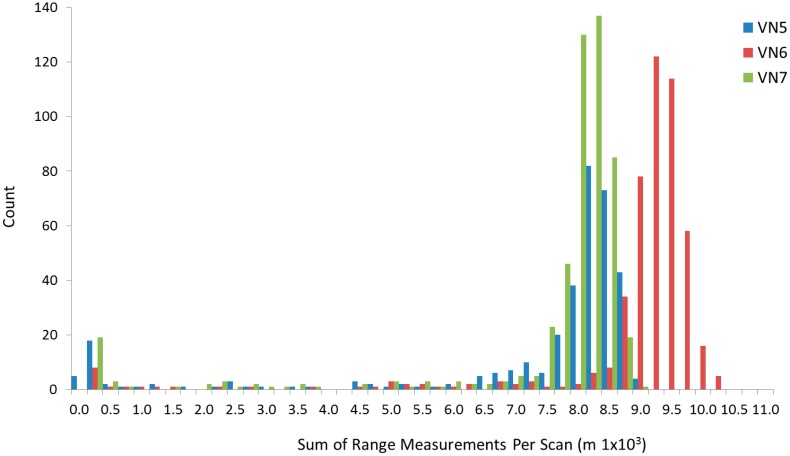
Frequency of total (summed) range measurements per scan.

Figure 5 shows the frequency of occurrence of summed range measurements within each scan. The histograms illustrate the differentiation between scan data of varying quality and form the basis of a threshold-based filtering system.

VEGNET IML scans were excluded from analysis if the sum of within-scan range measurements was less than 8000 m, 9000 m and 8100 m for IML instruments VN5, VN6 and VN7, respectively. The selected threshold values allow for monitoring of true changes in forest structure (e.g., a natural reduction in leaf area) without artificially constraining the amplitude of seasonal PAI variation. However, simple threshold-based data filtering may result in the exclusion of data during major change events such as pest or disease outbreak, tree fall or fire. As a result, the current method requires further development to improve the handling of such events. A dynamic threshold may be produced by regularly monitoring for persistent shifts in the distribution of total within-scan ranges and automatically re-calculating the PAI trends and PAVD profiles.

## 4. Results

**Figure 6.**
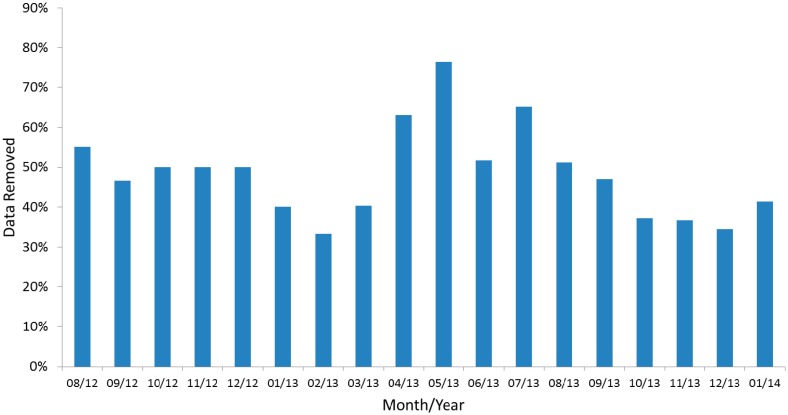
Percent of IML scans removed per month by data filtering processes.

Results from an 18 month deployment of VEGNET IML instruments are given to illustrate the method of data interpretation and indicative results at a monthly timescale. On average, 48% of data were excluded from analysis by the data filtering process. The minimum and maximum number of scans excluded in any one month was 33% and 76%, respectively. The percent of IML scans removed per month by data filtering processes is shown in Figure 6 for all three IML instruments combined.

A field visit on the 29 May 2012 revealed that instrument VN6 had been damaged by a falling tree branch resulting in the scan head prism being broken and water ingress from rainfall. Investigation of data files indicated that damage occurred on 12 May 2012. The scan head and GLM150 rangefinder were replaced on 10 June 2012. All VN6 data between 12 May and 9 June 2012 were excluded from analysis.

**Figure 7.**
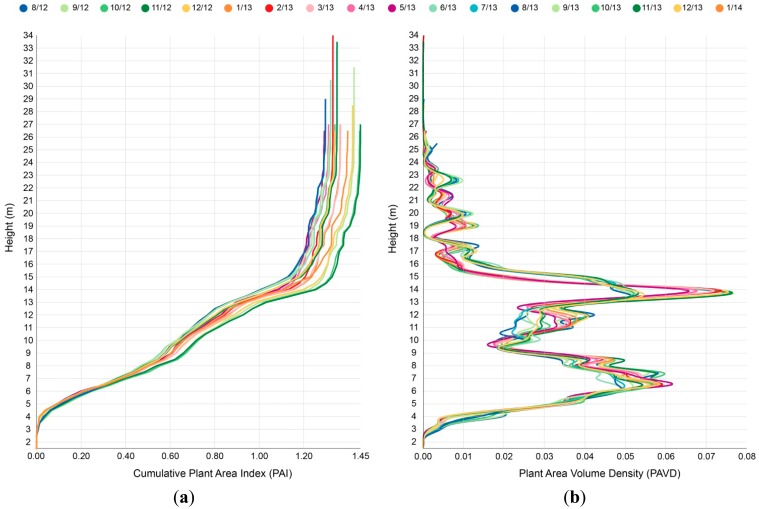
Apparent changes in vertical forest structure recorded by IML instrument number VN6. (**a**) Cumulative Plant Area Index. (**b**) Plant Area Volume Density. Different colors represent daily data aggregated at a monthly timescale. Common colors are used for each month across years to assist identification of seasonal trends. Blue, green, orange and purple colors represent the seasons of winter, spring, summer and autumn, respectively. Legend values are month/year.

Figure 7 shows the cumulative PAI and the vertical PAVD (plant density profile) for IML instrument number VN6 for the observation period August 2012 to February 2014. Data have been aggregated at a monthly timescale for clarity. The cumulative PAI profile, Figure 7a, shows high temporal consistency in the lower part of the canopy (up to approximately 8 m). These measurements are primarily of intransient structural elements such as tree stems and branches. Greater variability in the PAI profile appears as the height of measured targets increases. Greatest temporal variability occurs near the top of the canopy where the targets consist primarily of foliage and fine branches, and where the canopy will be more subject to movement from wind. Taking the derivative of the PAI profile produces the PAVD profile, Figure 7b. The PAVD profile highlights structural change both vertically and temporally. This information is central to IML instruments as a means of decoupling vegetation dynamics by vertical strata, thereby improving the calibration and validation of earth-observation data.

**Figure 8.**
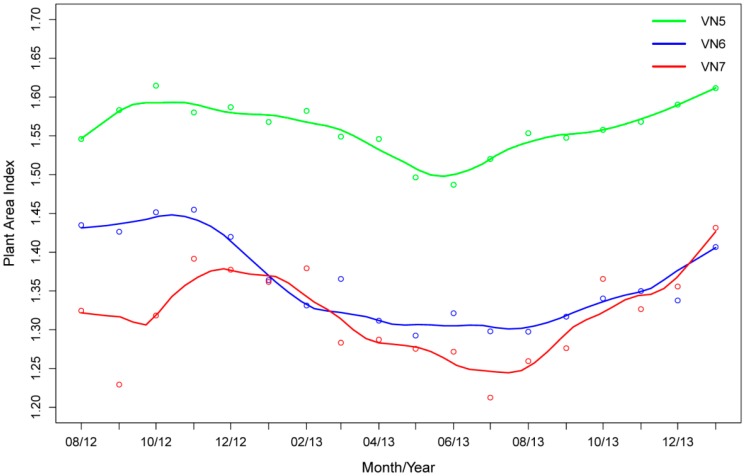
Long term PAI trend for the three IML instruments at the Rushworth Forest field site. Monthly PAI measurements are shown as circles. Trend lines fitted using spline interpolation.

Figure 8 shows the long-term variation in top-of-canopy PAI as estimated from the three VEGNET IML instruments within the Rushworth Forest study area. The figure shows that instrument VN5 is located in an area with overall higher PAI relative to instruments VN6 and VN7. The three IML instruments show broadly similar PAI trends over the 18-month observation period. Table 1 shows the long term mean PAI, range and variance for the three VEGNET IML instruments.

## 5. Discussion

Estimation of total PAI relies on effective discrimination of canopy elements and canopy gaps for a given view direction. Generation of vertical PAI profiles requires additional information on the height of canopy elements. The VEGNET IML assumes that invalid range measurements can be interpreted as gaps in the canopy. The relatively slow sample rate of the GLM150 laser (2–0.25 Hz) permits a visual validation of target type relative to range measurement. Based on such observations the majority of invalid range measurements can be attributed to gaps in the canopy. However, observation of the measurement process confirms that in some instances the laser beam is intercepted by vegetation but a range reading is not returned. Typically, this occurs when the target is at a distance greater than 60 m or when the target is moving due to wind.

Long-term validation of the VEGNET IML is required to understand these effects on PAI estimation and any bias that may result. It is possible that compensating errors may minimize bias. For example, canopy elements may leave the beam path due to wind, resulting in an apparent canopy gap. Conversely, canopy elements may enter the laser beam due to wind, resulting in beam interception along a previously unobscured beam path.

The vertical PAI and PAVD profiles from the IML field trial are consistent with field observations and structural characteristics of the Eucalypt Open Forest type. Figure 7b shows the canopy height within the scan range of instrument VN6 to be approximately 25 m tall, while peaks in canopy density occur at heights of approximately 6 m and 14 m. The mean long-term PAI values from each IML instrument (Table 1) are similar to LICOR LAI-2200 PAI estimates reported by [21] from a neighboring study site in the Rushworth State Forest. The long-term amplitude of PAI dynamics is difficult to validate due to limited information on PAI change at a high temporal frequency. The top-of-canopy PAI trends in Figure 8 are broadly consistent across all three instruments, suggesting reliable instrument response to observed structural change. Key features of the trend are a decrease in PAI from December to July (summer to winter), followed by an increase in PAI from July to December.

Rather than the observed continuing decline of PAI during winter, it was expected that the strongest decline would occur during summer months from December to February, which is a common response of temperate eucalypt forests resulting from growth and physiological stress [22]. Autumn and winter rainfall would normally bring an increase in PAI followed by a flush of new growth in early spring as temperatures increase. Inter-annual variability in growing conditions does not permit a definitive validation of VEGNET IML relative to previously observed seasonal patterns. In particular, observations from the on-site weather station showed that the study area received only 146 mm of rainfall from January to July, less than half the rainfall relative to long term (1970–2012) meteorological records from regional stations. A drought statement from Australia's National Climate Center [23] identified the study area as having rainfall in the lowest 5th percentile on record in the 9 months from October 2012 to June 2013. The State of Victoria also recorded its warmest winter on record in 2013 [24]. Increased leaf litter fall during stressful growing conditions may explain the winter decline in PAI as observed by all three IML instruments. Above-average rainfall was recorded at the study site for the month of August 2013, helping to sustain the new growth trend observed through spring. The performance of the VEGNET IML will become clearer as additional years of data are acquired.

The VEGNET IML instrument has satisfactorily achieved long-term, high temporal frequency monitoring of forest structural dynamics. In this study, IML data are treated aspatially for producing PAI and PAVD profiles. All gap and range measurements for each scan are combined to produce a “plot scale” measurement, where the plot radius is approximately twice the canopy height. The low spatial detail captured by the VEGNET IML design does not support monitoring structural change at an individual tree scale. However, the IML instruments are suitable for monitoring large plots (up to approximately 1 ha) on a daily timescale, which is spatially and temporally compatible with earth-observation sensors.

## 6. Conclusions

LAI is a difficult vegetation parameter to quantify [4]. Vastly different approaches have been employed for estimating LAI measurement and the method selected can influence the value retrieved [25–28]. A key strength of IML is the potential to monitor *relative* changes in LAI from a fixed location with consistent methodology and insensitivity to (solar) illumination conditions [29]. So, while uncertainty and variability in absolute measurement of PAI may persist among measurement techniques and with the validity of underlying assumptions, there is a new generation of inexpensive laser rangefinder that is enabling *in-situ* monitoring in a manner that was not possible or practical until very recently. The VEGNET IML is the first instrument of its kind to exploit this new technology for automated forest monitoring. A rigorous validation of the VEGNET IML instrument in diverse forest types is part of an ongoing research program.

In addition to providing a stable method for the measurement of PAI, the IML provides additional information about the vertical distribution of PAI. This information is key to understanding the vertical distribution of the canopy light field and how this may affect growth. In terms of monitoring forest health, the vertical profiles measured by the IML have the capability to not only provide an indication of disturbance but where in the canopy (which strata) this disturbance is occurring.

The inexpensive nature of the IML allows for the development of wireless sensor networks (VEGNET). These networks are the strength of IML instruments, being able to monitor the condition of forests over broad regions for the purpose of meeting State based reporting obligations and managing forest disturbance in a timely manner.

## Figures and Tables

**Table 1.** PAI statistics (mean, maximum, minimum and range) for the three IML instruments over the 18 month observation period.

**Instrument**	**Mean PAI**	**Maximum PAI**	**Minimum PAI**	**PAI Range**	**PAI Range (%)**
VN5	1.56	1.61	1.49	0.13	8
VN6	1.36	1.45	1.29	0.16	12
VN7	1.32	1.43	1.21	0.22	17
